# Impact of Long-Acting Somatostatin Analogues on Glucose Metabolism in Acromegaly: A Hospital-Based Study

**DOI:** 10.1155/2018/3015854

**Published:** 2018-04-26

**Authors:** Ming Shen, Meng Wang, Wenqiang He, Min He, Nidan Qiao, Zengyi Ma, Zhao Ye, Qilin Zhang, Yichao Zhang, Yeping Yang, Yanjiao Cai, Yakupujiang ABuDuoReYiMu, Yun Lu, Bin Lu, Xuefei Shou, Yongfei Wang, Hongying Ye, Yiming Li, Shiqi Li, Yao Zhao, Xiaoyun Cao, Zhaoyun Zhang

**Affiliations:** ^1^Department of Neurosurgery, Huashan Hospital, Fudan University, Shanghai 200040, China; ^2^Department of Endocrinology and Metabolism, Huashan Hospital, Fudan University, Shanghai 200040, China; ^3^Department of Endocrinology and Metabolism, The Second People's Hospital of Kashi, Xinjiang Uygur Autonomous Region 844000, China; ^4^Department of Nuclear Medicine, Huashan Hospital, Fudan University, Shanghai 200040, China

## Abstract

**Purpose:**

To evaluate the change in glucose tolerance in treatment-naïve patients with acromegaly after administration of SSA and to identify predictive factors of glucose impairment during SSA therapy.

**Methods:**

Oral glucose tolerance testing (OGTT) was performed on 64 newly diagnosed and treatment-naïve patients with acromegaly both at pretreatment and 3 months after initiation of treatment with long-acting SSA. Insulin resistance (IR) was assessed by homeostatic model assessment- (HOMA-) IR and IS_OGTT_. Insulin secretion was assessed by HOMA-*β*, INS_0_/BG_0_, IGI (insulinogenic index), IGI/IR, ISSI2, and AUC_INS_/AUC_BG_. Receiver-operating characteristic (ROC) curves were generated to determine the optimal cutoffs to predict the impact of SSA on glucose metabolism.

**Results:**

Pretreatment, 19, 24, and 21 patients were categorized as having normal glucose tolerance (NGT), impaired glucose tolerance (IGT), and diabetes mellitus (DM), respectively. Posttreatment, IR, represented by IS_OGTT_, was significantly improved in all 3 groups. Insulin secretion, represented by HOMA-*β*, declined in the NGT and IGT groups, but was unaltered in the DM group. The glucose tolerance status deteriorated in 18 (28.1%) patients, including 13 patients in the NGT group and 5 patients in the IGT group. Deterioration was associated with lower baseline BG_120_ (plasma glucose 120 min post-OGTT), less reduction of growth hormone (GH), and greater reduction of insulin secretion after SSA therapy. BG_120_ greater than 8.1 mmol/l provided the greatest sensitivity and specificity in predicting the stabilization and/or improvement of glucose tolerance status after SSA treatment (PPV 90.7%, NPV 66.7%, *p* < 0.001).

**Conclusions:**

The deterioration of glucose metabolism induced by SSA treatment is caused by the less reduction of GH and the more inhibition of insulin secretion, which can be predicted by the baseline BG_120_ during OGTT.

## 1. Introduction

Acromegaly is an insidious disease associated with a 1.72 times increased mortality risk [1]. Cardiovascular, respiratory, and metabolic complications are the main causes of death in acromegaly. Disturbances of carbohydrate metabolism are the major type of metabolic disorder [2]. Overt type 2 diabetes mellitus is reported in 19–56% and impaired glucose tolerance (IGT) in 16–46% of patients with acromegaly [3]. GH-mediated insulin resistance (IR) is the major cause of impaired glucose metabolism in active acromegaly [4].

Although transsphenoidal surgery is the first-line therapy for GH-secreting adenomas, for those who are not in remission after surgery or for whom surgery is contraindicated, long-acting somatostatin analogues (SSA) are generally considered to be first-line therapy [5]. However, the impact of SSA on glucose metabolism has not been fully elucidated and previous results from small series are conflicting [6–9]. This may be due to the fact that SSA inhibits GH and glucagon secretion while also suppressing the release of insulin [10, 11]. The aim of our study was to investigate the effects of SSA on glucose homeostasis and to determine whether there are any variables that could predict the influence of SSA on glucose metabolism in patients with active acromegaly.

## 2. Subjects and Methods

### 2.1. Patients

This was a retrospective study of prospectively obtained data from patients seen between July 2012 and August 2014 at a tertiary referral center in the East of China. Sixty-four newly diagnosed and untreated patients with acromegaly (38 females and 26 males, mean age 41.7 ± 13.0 years) were recruited. Clinical and biochemical findings of the patients are summarized in Supplementary Tables 1–4. The diagnosis of active disease was based on the clinical features of acromegaly, failure of GH suppression to below 1 *μ*g/l in response to a 75 g oral glucose tolerance test (OGTT), plasma IGF-1 levels above the age-appropriate reference range, and radiological evidence of a pituitary tumor. The mean GH (GH_m_) was obtained as the average level of 5 samples drawn within a 2 h period (every 30 min from 0700 to 0900 h) [12]. Before and after SSA treatment, glycosylated hemoglobin (HbA_1c_) was obtained. Glucose tolerance was evaluated by OGTT. Briefly, after an overnight fasting, blood samples were drawn for baseline blood glucose (BG) and insulin (INS). Then, 75 g of glucose was administered orally. Sampling for BG and insulin was performed 30, 60, 120, and 180 min later. Three months after initiation of long-acting SSA treatment, octreotide LAR, 20 mg every 4 weeks (*N* = 42), and lanreotide SR, 40 mg every 2 weeks (*N* = 22), patients were reevaluated. The diagnosis of type 2 diabetes mellitus or impaired glucose tolerance was made according to World Health Organization criteria [13].

Informed consent was obtained by each individual. Our study was approved by the ethics committee at our hospital and was performed in accordance with the ethical standards laid down in the 1964 Declaration of Helsinki and its later amendments.

### 2.2. Evaluation of Insulin Resistance and *β*-Cell Function

Homeostatic model assessment (HOMA) (including HOMA-IR and HOMA-*β*) was used to estimate insulin resistance (IR) and *β*-cell function [14]. Insulin sensitivity was also assessed by calculating IS_OGTT_ (the OGTT insulin sensitivity index) [15]. INS_0_/BG_0_, IGI (insulinogenic index), IGI/IR, and ISSI2 (the OGTT insulin secretion sensitivity index-2) were also used to estimate *β*-cell function [14, 16–22]. The areas under the curve of glucose (AUC_BG_) and insulin (AUC_INS_) during OGTT were calculated using the trapezoidal rule [9, 23]. AUC_INS_/AUC_BG_, which is an indicator of insulin secretion, was also calculated [20].

### 2.3. Abbreviated Variables and Formulas

BG_0_ was the baseline blood glucose value during the OGTT. BG_30_, BG_60_, BG_120_, and BG_180_ were the blood glucose values from 30 min to 180 min during the OGTT. INS_0_, INS_30_, INS_60_, INS_120_, and INS_180_ were the insulin values from basal to 180 min during the OGTT. BG_mean_ and INS_mean_ represent the mean insulin and glucose concentrations during the OGTT. AUC_BG_ = (BG_0_ + BG_30_) × 15 + (BG_30_ + BG_60_) × 15 + (BG_60_ + BG_120_) × 30 + (BG_120_ + BG_180_) × 30. AUC_INS_ = (INS_0_ + INS_30_) × 15 + (INS_30_ + INS_60_) × 15 + (INS_60_ + INS_120_) × 30 + (INS_120_ + INS_180_) × 30. HOMA-IR = (BG_0_ × INS_0_)/22.5. HOMA-*β* = (20 × INS_0_)/(BG_0_ – 3.5) × 100%. IS_OGTT_ (the OGTT insulin sensitivity index) = 10,000/SQRT (BG_0_ × INS_0_ × BG_mean_ × INS_mean_). IGI (insulinogenic index) = (INS_30_ − INS_0_)/(BG_30_ − BG_0_) = △INS_30_/△BG_30_. IGI/IR = IGI/HOME-IR. ISSI2 (the OGTT insulin secretion sensitivity index-2) = (AUC_INS_/AUC_BG_) × IS_OGTT_.

### 2.4. Biochemical Measurements

GH was measured by a two-site chemiluminescent immunometric assay (AutoDELFIA® hGH, PerkinElmer Life and Analytical Sciences, Wallac Oy), intra-assay CV: 5.3–6.5%, interassay CV: 5.7–6.2%, and sensitivity: up to 0.01 *μ*g/l (0.026 mU/l).

IGF-1 was measured with the IMMULITE 2000 solid-phase, enzyme-labeled chemiluminescent immunometric assay (Siemens Healthcare Diagnostic Products Limited, UK); normal age-appropriate ranges are as follows: 1–6 years: 49–327 *μ*g/l; 7–11 years: 57–551 *μ*g/l; 12–13 years: 143–850 *μ*g/l; 14–16 years: 220–996 *μ*g/l; 17–18 years: 163–731 *μ*g/l; 19–20 years: 127–483 *μ*g/l; 21–35 years: 115–358 *μ*g/l; 36–50 years: 94–284 *μ*g/l; >50 years: 55–238 *μ*g/l; intra-assay CV: 2.3–3.5%; interassay CV: 7.0–7.1%; and sensitivity: 20 *μ*g/l. IGF-1 index = IGF − 1/upper limit of normal range (ULN) [24].

Insulin was measured by chemiluminescence immunoassay (ADVIA Centaur XP, Siemens, USA). BG was measured by a Hitachi 7600 Biochemical Analyzer (Tokyo, Japan). HbA_1c_ was detected with high-performance liquid chromatography (Tosoh HLC-723 G8 HPLC Analyzer, Japan).

### 2.5. Statistics Analysis

Data are presented as mean ± SD (or median with interquartile range) for continuous variables normally (or not normally) distributed, respectively, and as frequency for categorical variables. Normality was tested using the Kolmogorov-Smirnov test. The change of variables between pre- and post-SSA treatment within one group was compared using the paired *t*-test when data distribution was normal or by the Wilcoxon rank-sum (Mann–Whitney) test when variables were not normally distributed. One-way ANOVA with LSD post hoc analysis (or the Kruskal-Wallis test followed by Bonferroni post hoc test) was used for comparisons among multiple groups. For categorical variables, differences were analyzed by the chi-square test. Univariate regression analysis was performed, and Spearman rank correlation coefficients are reported. After construction of receiver-operating characteristic (ROC) curves, Youden indices were calculated to determine the optimal cutoffs for variables to predict the change in glucose metabolism after SSA treatment (sensitivity, specificity, PPV, and NPV). Statistical analysis was performed with SPSS 16.0 statistical software. A two-tailed *p* value < 0.05 was considered significant.

## 3. Results

### 3.1. Baseline Characteristics among NGT/IGT/DM Groups

Pretreatment, patients were categorized into three groups: normal glucose tolerance (NGT) group (19 patients, 8 females/11 males), impaired glucose tolerance (IGT) group (24 patients, 15 females/9 males), and diabetes mellitus (DM) group (21 patients, 15 females/6 males). 8 patients in the DM group were known to have diabetes and were treated with oral antidiabetic drugs prior to taking part in this study. For these patients, OGTT was only performed when fasting plasma glucose (FPG) was below 8 mmol/l (previously diagnosed diabetic patients with FPG above 8 mmol/l was excluded from this study). The other 13 diabetic patients were diagnosed at baseline OGTT along with the diagnosis of acromegaly. During the study, 10 patients were treated with oral antidiabetic drugs and 11 patients were given advice about lifestyle/dietary modifications. The baseline characteristics of the three groups are shown in Supplementary Table 5. Age, body mass index (BMI), GH_m_, IGF-1 index, and HOMA-IR did not differ significantly among the three groups. HbA_1c_ was higher in the DM group than in the NGT and IGT groups, while HOMA-*β* was significantly lower in the DM group than in the other two groups.

No difference was found between females and males in age, BMI, HbA1c, GH_m_, FPG, and BG_120_. Females had significantly higher FPI, INS_120_, HOME-*β*, INS_0_/BG_0_, and HOMA-IR, with lower IS_OGTT_ and lower IGF-1 index, than males had (Supplementary Table 6). Thus, females were prone to higher insulin resistance and higher *β*-cell function than males were.

### 3.2. Effect of SSA Treatment on BG and HbA_1c_ Levels

Compared to pretreatment, HbA_1c_ dropped significantly within the DM group (8.35 ± 2.47 versus 6.88 ± 1.00%, *p* = 0.015) after SSA treatment. In the entire cohort, NGT, and IGT groups, HbA_1c_ showed no change from pretreatment to posttreatment (Table 1).

Compared to pretreatment, FPG increased significantly in the entire cohort, NGT, and IGT groups after SSA treatment. However, in the DM group, no changes were detected from pretreatment to posttreatment. From before to after SSA treatment, BG_120_ increased in the NGT group and decreased in the DM group, while it was unaltered in the entire cohort and IGT group (Table 1).

### 3.3. Effect of SSA Treatment on Plasma Insulin Levels during OGTT

Compared to pretreatment, the posttreatment levels of fasting plasma insulin (FPI) declined in the group as a whole and in NGT and IGT groups. However, no change was detected within the DM group from pretreatment to posttreatment. Compared to pretreatment, after SSA treatment, INS_120_ decreased in the group as a whole and in the IGT group, but remained unaltered in the NGT and DM groups (Table 1).

### 3.4. Effect of SSA Treatment on Insulin Resistance

After SSA treatment, HOMA-IR significantly decreased within the group as a whole, and in the NGT and IGT groups, but not in the DM group. Moreover, IS_OGTT_ significantly increased in the group as a whole, as well as in the NGT, IGT, and DM groups (Table 1). After SSA treatment, HOMA-IR significantly decreased, while IS_OGTT_ significantly increased, in both females and males (Supplemental Table 7).

### 3.5. Effect of SSA Treatment on Insulin Secretion

In the group as a whole and in the IGT group, there was a significant decline in *β*-cell function, including HOMA-*β*, INS_0_/BG_0_, IGI, IGI/IR, and AUC_INS_/AUC_BG_ after SSA treatment. However, no significant change was observed in ISSI2. In the NGT group, all variables reflective of *β*-cell function declined. However, in the DM group, no change was observed in any variables reflective of insulin secretion (Table 1). In females, all variables reflective of *β*-cell function declined except AUC_INS_/AUC_BG_. In males, all variables reflective of *β*-cell function declined except ISSI2 (Supplementary Table 7).

### 3.6. Effects of SSA Treatment on Glucose Tolerance

**Figure 1:**
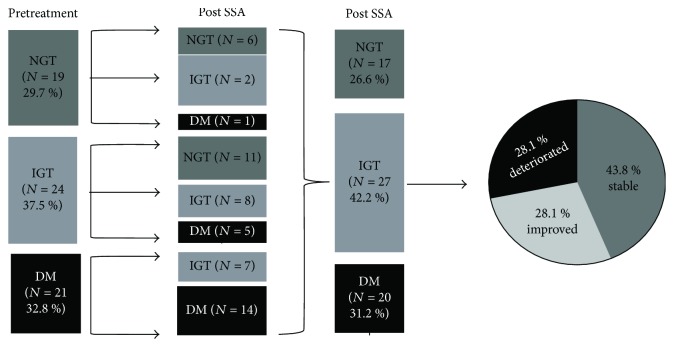
Flowchart of prevalence of NGT, IGT, and DM at pretreatment and after SSA treatment, and the change of glucose metabolism status after SSA therapy. NGT: normal glucose tolerance; IGT: impaired glucose tolerance; DM: diabetes mellitus.

At the baseline, 29.7% (19/64) of patients had NGT, 37.5% (24/64) had IGT, and 32.8% (21/64) had DM. After SSA treatment for 3 months, 26.6%, 42.2%, and 31.2% of the patients, respectively, were categorized as NGT, IGT, and DM (Figure 1). After SSA treatment, in the NGT group (*n* = 19), 31.5% maintained the status quo, while 63.2% developed IGT and 5.3% became diabetic. In the IGT group (*n* = 24), 45.8% of the patients became NGT, 33.4% remained unchanged, and 20.8% progressed to diabetes. In the DM group (*n* = 21), 66.7% continued to have diabetes mellitus while 33.3% improved to IGT. In summary, after SSA treatment, the distribution of glucose metabolism status was as follows: 43.8% (28/64) patients were stable, 28.1% (18/64) of the subjects improved, and 28.1% (18/64) of the subjects deteriorated (Figure 1).

After SSA therapy, subjects were classified into 3 groups according to the change of glucose tolerance category: Improved (*n* = 18, from IGT to NGT, from DM to IGT, or from DM to NGT), Stable (*n* = 28, from NGT to NGT, from IGT to IGT, or from DM to DM), and Deteriorated (*n* = 18, from NGT to IGT, from NGT to DM, or from IGT to DM). The baseline characteristics of these 3 groups are shown in Table 2. Patients in the Stable group were older than those in the other two groups (*p* = 0.049). The baseline BG_120_ levels were significantly lower in the Deteriorated group than in the other two groups (*p* < 0.001).

The changes in glucose metabolism-related variables after SSA treatment are shown in Table 3. The reduction of GH_m_ was much less in the Deteriorated group than in the other two groups (*p* = 0.021). The reduction of HOMA-*β* was greater in the Deteriorated group than in the Stable group (*p* = 0.043) and Improved group (*p* = 0.046).

Patients were further divided into biochemically controlled (*n* = 16, posttreatment GH levels < 2.5 *μ*g/l) group and uncontrolled (*n* = 35, posttreatment GH levels ≥ 2.5 *μ*g/l) group based on posttreatment GH levels. As shown in Supplementary Table 8, We found a trend toward a decrease on HbA_1c_ (6.09 ± 1.32 versus 5.81 ± 0.64%), FPG (5.68 ± 1.75 versus 5.53 ± 0.45 mmol/l), and BG_120_ (8.75 versus 7.85 mmol/l) in the controlled group. As for the change in insulin resistance and secretion, we found that after treatment, insulin resistance, represented by IS_OGTT_, was significantly improved in both groups. And all variables reflective of insulin secretion except ISSI2 declined in both groups (Supplementary Table 8).

### 3.7. Correlation Studies

**Figure 2:**
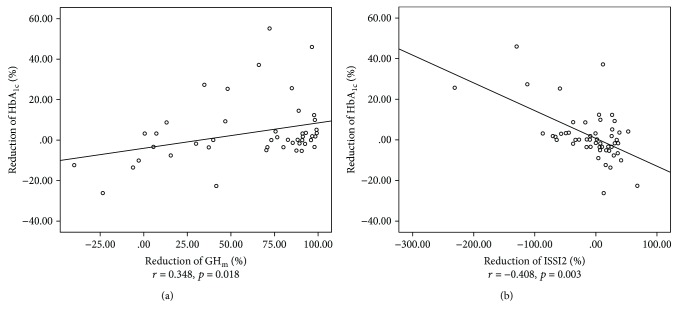
The reduction of HbA_1c_ was positively correlated with the reduction of GH_m_ (a) and negatively correlated with the reduction of ISSI2 (b) after SSA treatment in the entire cohort. (a) The correlation between the reduction of HbA_1c_ and the reduction of GH_m_. (b) The correlation between the reduction of HbA_1c_ and the reduction of ISSI2. Correlation coefficients and *p* values were shown for each correlation.

In the group as a whole, the reduction in HbA_1c_ positively correlated with the reduction in GH_m_ (*r* = 0.348, *p* = 0.018, Figure 2(a)) and negatively correlated with the reduction of ISSI2 (*r* = −0.408, *p* = 0.003, Figure 2(b)), IGI (*r* = −0.294, *p* = 0.032), and IGI/IR (*r* = −0.273, *p* = 0.048) after SSA treatment (Supplementary Table 9).

### 3.8. The Predictive Value of Baseline BG_120_ for the Effect of SSA Treatment on Glucose Metabolism

**Figure 3:**
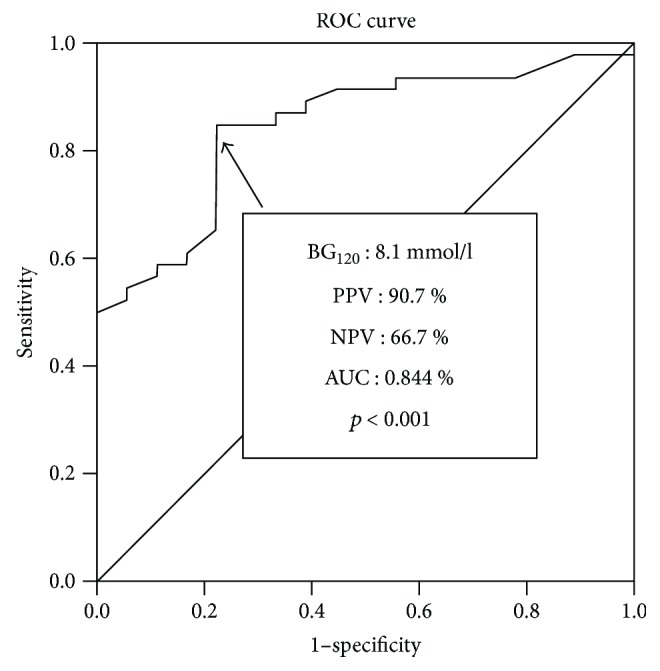
ROC curve analysis of pretreatment BG_120_ during OGTT in predicting the stability and/or improvement of glycemic status after SSA treatment in the entire cohort. The central line indicates neutrality, and the arrow shows the baseline BG_120_ 8.1 mmol/l during the OGTT with a PPV of 90.7% and a NPV of 66.7% (AUC = 0.844, *p* < 0.001).

ROC curve analysis was performed to further estimate the predictive value of BG_120_ on the change of glucose tolerance status. The cutoff value of baseline BG_120_ was 8.1 mmol/l which demonstrated the greatest sensitivity and specificity in predicting the stability and/or improvement of glycemic status after SSA treatment, with a PPV of 90.7% and a NPV of 66.7% (sensitivity 84.8%, specificity 77.8%, AUC = 0.844, *p* < 0.001, Figure 3).

Patients were categorized into two groups according to BG_120_ at baseline: group A (BG_120_ greater than 8.1 mmol/l) and group B (BG_120_ less than 8.1 mmol/l). First, we compared these two groups at baseline. We found that IGI (*p* = 0.001), IGI/IR (*p* < 0.001), and ISSI2 (*p* < 0.001) were higher in group B than in group A (Supplementary Table 10). Second, the changes in variables after SSA treatment were analyzed (Supplementary Table 11). We found that the reduction of GH_m_ was less in group B than in group A (*p* = 0.019), while the reduction of HOMA-*β*, IGI, IGI/IR, and ISSI2 was more in group B than in group A (*p* = 0.037, 0.002, 0.008, and 0.046, resp.).

## 4. Discussion

In the present study, we demonstrated that the change in glucose metabolic status after SSA therapy strongly correlated with the baseline status of glucose metabolism in patients with acromegaly. FPG rose in both NGT and IGT groups, but remained stable in the DM group. BG_120_ increased in the NGT group, stabilized in the IGT group, and decreased in the DM group. Insulin resistance was improved in all 3 groups, while insulin secretion declined in the NGT and IGT groups and was unchanged in the DM group. The glucose tolerance status was improved in 28.1% patients, deteriorated in 28.1% patients, and stabilized in 43.8% patients. Deterioration was associated with lower baseline BG_120_, less reduction in GH_m_, and a greater reduction in insulin secretion after SSA therapy. The cutoff value of BG_120_ (8.1 mmol/l) at baseline predicted the stabilization and/or improvement of glucose metabolism during SSA treatment.

The impact of SSA on glucose metabolism has been studied, but the results are conflicting [6–9]. Several studies have reported no change of glucose levels after SSA treatment [6, 25]. A meta-analysis also indicated that SSA might have an overall minor impact on glucose homeostasis in patients with acromegaly [26]. However, others found that SSA significantly aggravated glucose tolerance in patients with acromegaly [11, 27–29], thus mandating glucose monitoring during SSA therapy. Interestingly, Ho et al. even reported that SSA has beneficial effects on carbohydrate metabolism in patients with acromegaly and glucose intolerance [30]. In our study, the predominant pattern of change in glucose tolerance status was deterioration in the baseline NGT group, stabilization in the baseline IGT group, and amelioration in the baseline DM group. And the influence of SSA on glucose metabolism was not gender specific, although females were prone to have higher insulin resistance and higher *β*-cell function at baseline, which was highly consistent with the study of Ciresi et al. [31]. These data suggest that depending on the glucose tolerance status at baseline, SSA has distinct effects on the glucose metabolism in patients with acromegaly. This might partially explain the conflicting results from previous studies which had patients with different glucose tolerance status.

Recently, pasireotide was approved for acromegaly and showed more efficacy in controlling GH and IGF-1 levels [32]. As for the effects on glucose metabolism, a head-to-head study has reported that compared with octreotide LAR, hyperglycemia-related adverse events were more common with pasireotide [33].

The change in glucose metabolism correlated strongly with the change of insulin resistance and insulin secretion after SSA treatment [34]. Ronchi et al. found that HOMA-IR significantly declined during SSA treatment [9]. Baldelli et al. found that insulin resistance was improved but the insulin secretion was 30 minutes delayed after 6 months of SSA therapy [27]. However, Steffin et al. found that SSA decreased *β*-cell function without affecting insulin resistance [35]. In the present study, we used not only HOMA but also various derivatives of the OGTT to evaluate insulin sensitivity and *β*-cell function. For insulin sensitivity, HOMA-IR decreased in the NGT and IGT groups and remained unaltered in the DM group, while IS_OGTT_, another major parameter reflecting insulin resistance, improved in all groups. Matsuda et al. first developed the IS_OGTT_ index and proved IS_OGTT_ to be a reasonable and better approximation of whole-body insulin sensitivity in patients with diabetes mellitus than HOMA [15]. This might be applicable to patients with acromegaly. Variables reflective of *β*-cell function, such as HOMA-*β*, INS_0_/BG_0_, IGI/IR, and IGI, declined in both NGT and IGT groups, but remained unchanged in the DM group. The above results showed SSA decreased insulin secretion in NGT and IGT groups, but had no effect in the DM group.

Excess GH levels led to insulin resistance in both NGT and DM patients, and SSA therapy could significantly reduce GH levels, resulting in the decrease of insulin resistance both in NGT and DM groups. Meanwhile, insulin secretion was decreased after SSA treatment in the NGT group, but was not compromised in the DM group. Thus, the glucose metabolic status was generally improved after SSA administration in the DM group due to the alleviated degree of insulin resistance without compromise of insulin secretion. But in the NGT group, the glucose metabolic status might even deteriorate if the reduction of insulin secretion overcomes the improvement of insulin resistance. This may be the potential underlying mechanism for the different effects of SSA on glucose metabolism in patients with NGT and patients with DM.

Several studies revealed factors associated with the SSA-induced changes in glucose tolerance status. Koop et al. stated that female patients and those with higher baseline insulin levels were more likely to develop DM during SSA therapy [11]. Ho et al. found that improvement in glucose tolerance status was dependent on pretreatment BG concentrations [30]. Colao et al. found that deterioration of glucose metabolism was correlated with increased BMI, uncontrolled acromegaly during SSA therapy, and abnormal glucose tolerance at baseline [28, 29]. In the present study, we showed that the deterioration of glucose tolerance was associated with less reduction of GH and greater reduction in insulin secretion after SSA therapy. In addition, the reduction of HbA_1c_ was positively correlated with the reduction of GH_m_ and negatively correlated with the reduction of insulin secretion. Interestingly, we found that SSA administration can significantly improve insulin resistance with a compromise in insulin secretion, in patients with both biochemically controlled (posttreatment GH levels < 2.5 *μ*g/l) and uncontrolled (posttreatment GH levels ≥ 2.5 *μ*g/l) acromegaly, which was similar with Giordano et al. [36]. Some discrepancy (e.g., IGI) may be related to the different races and duration of SSA treatment (3 months in our study, ≥12 months in literature) between studies. When exploring potential baseline predictors, we found that the baseline BG_120_ was significantly lower in patients whose glucose status deteriorated. Furthermore, for the first time, we generated ROC curves to obtain the most sensitive and specific cutoff values which predicted the change of glucose metabolism after SSA therapy. We showed that when the baseline BG_120_ was higher than 8.1 mmol/l, there was a 90.7% chance of stabilized and/or improved glucose tolerance status. However, when the baseline BG_120_ was lower than 8.1 mmol/l, there was a 66.7% chance of deterioration in glucose tolerance status.

To explore the potential mechanism, we examined the difference between patients with baseline BG_120_ above 8.1 mmol/l and those with baseline BG_120_ below 8.1 mmol/l. Interestingly, we found that patients with baseline BG_120_ below 8.1 mmol/l had less of a reduction in GH_m_ and a greater reduction in *β*-cell function. Less reduction of GH_m_ led to less improvement in insulin resistance in patients with baseline BG_120_ below 8.1 mmol/l. In addition to less improvement in insulin resistance, patients with baseline BG_120_ below 8.1 mmol/l had a greater reduction in insulin secretion, which indicated that there was more chance of deteriorating glucose tolerance status after SSA treatment in these subjects. Thus, vice versa, baseline BG_120_ higher than 8.1 mmol/l after OGTT may be considered as a beneficial predictive factor for glucose metabolism during SSA treatment. This seemed to be discordant with a previous study indicating that baseline glucose status was one of the major predictors of changing glucose status [29]. But actually, in our study, the percentage of improved glucose metabolism in the IGT group tended to be more than in the DM group [45.8% (11/24) versus 33.3% (7/21), *p* = 0.393], which was consistent with the study of Colao et al.

The limitation of the current study is that this study is not a blinded study from a patient's point of view and patients who are diagnosed with diabetes mellitus or impaired glucose tolerance at pretreatment assessment may have lifestyle/dietary modification, which may have had an impact on the glucose metabolism results in the follow-up assessment.

In conclusion, the impact of SSA on the change in glucose metabolic status, insulin resistance, and *β*-cell function depends on the pretreatment glucose metabolism status in patients with acromegaly. Deterioration is associated with lower baseline BG_120_, the less of a reduction in GH_m_, and a greater reduction in insulin secretion after SSA therapy. BG_120_ during OGTT can predict the impact of SSA treatment on glucose metabolism.

## Figures and Tables

**Table 1:** Changes of variables in NGT, IGT, and DM groups from pretreatment to after SSA treatment.

	The entire cohort (*n* = 64)			NGT (*n* = 19)			IGT (*n* = 24)			DM (*n* = 21)		
	Pre-SSA	Post-SSA	*p* value	Pre-SSA	Post-SSA	*p* value	Pre-SSA	Post-SSA	*p* value	Pre-SSA	Post-SSA	*p* value
HbA_1c_ (%)	5.80 (5.60~6.40)	6.00 (5.50~6.50)	0.256	5.64 ± 0.27	5.78 ± 0.38	0.083	5.72 ± 0.37	5.73 ± 0.45	0.914	8.35 ± 2.47	6.88 ± 1.00	0.015^∧^
FPG (mmol/l)	5.50 (5.10~6.20)	5.90 (5.40~6.38)	0.015^∧^	5.08 ± 0.41	5.63 ± 0.55	<0.001^∧^	5.52 ± 0.55	5.88 ± 0.68	0.025^∧^	7.29 ± 2.22	6.71 ± 1.89	0.251
BG_120_ (mmol/l)	8.90 (7.23~12.88	8.70 (7.40~12.08)	0.477	6.23 ± 0.99	8.37 ± 1.50	<0.001^∧^	8.97 ± 0.86	8.28 ± 2.48	0.233	16.00 ± 3.24	13.80 ± 4.30	0.002^∧^
FPI (mU/l)	14.80 (11.00~25.18)	10.05 (6.30~20.08)	<0.001^∧^	14.10 (9.70~24.10)	8.40 (4.40~18.20)	0.001^∧^	19.20 (13.37~28.38)	16.45 (7.03~19.55)	0.001^∧^	12.40 (5.45~20.60)	10.00 (5.00~21.30)	0.205
INS_120_ (mU/l)	93.85 (44.60~191.10)	63.20 (34.03~129.33)	0.001^∧^	66.90 (44.70~148.90)	74.90 (56.80~131.20)	0.777	185.75 (115.50~298.31)	63.30 (41.05~192.70)	0.004^∧^	44.40 (21.50~106.00)	36.20 (19.25~68.00)	0.071
HOMA-IR	4.27 (2.78~6.64)	2.39 (1.57~4.78)	<0.001^∧^	3.32 (2.20~6.01)	1.94 (0.95~4.45)	0.014^∧^	4.78 (3.28~7.04)	2.74 (2.07~4.73)	0.007^∧^	4.08 (2.02~7.13)	2.44 (1.72~7.37)	0.073
IS_OGTT_	41.74 (25.72~67.28)	69.70 (42.23~117.02)	<0.001^∧^	50.21 (28.35~70.91)	87.07 (46.72~145.93)	0.002^∧^	31.90 (23.05~41.74)	54.34 (34.19~111.57)	<0.001^∧^	49.74 (27.07~86.96)	81.46 (42.15~124.76)	0.044^∧^
HOMA-*β* (%)	165.69 (83.85~255.24)	90.57 (52.98~169.30)	<0.001^∧^	202.61 (137.50~283.53)	91.58 (60.00~154.07	<0.001^∧^	188.50 (140.87~290.86)	96.77 (66.34~192.76	<0.001^∧^	73.75 (27.79~137.22)	77.50 (36.84~177.09)	0.394
INS_0_/BG_0_	2.75 (1.83~4.63)	1.73 (1.05~3.28)	<0.001^∧^	2.74 (1.90~4.63)	1.61 (1.00~3.31)	<0.001^∧^	3.50 (2.61~5.02)	1.80 (1.26~3.69)	<0.001^∧^	1.93 (0.70~3.05)	1.72 (0.74~3.20)	0.305
IGI	20.66 (6.16~45.11)	4.45 (1.89~10.21)	<0.001^∧^	34.20 (16.97~60.00)	4.74 (2.80~17.50)	0.002^∧^	32.69 (18.68~47.53)	9.20 (4.64~17.52)	<0.001^∧^	2.15 (1.17~7.55)	1.83 (0.97~3.14)	0.117
IGI/IR	4.81 (1.67~9.16)	2.19 (0.91~4.42	<0.001^∧^	9.48 (5.11~16.07)	4.09 (1.30~7.34)	0.007^∧^	5.19 (3.46~7.65)	3.42 (2.26~5.01)	0.026^∧^	0.71 (0.35~1.85)	0.99 (0.30~1.62)	0.360
ISSI2	457.88 (259.97~586.72)	378.35 (248.50~509.10)	0.242	587.42 (511.92~710.87)	484.22 (341.33~734.40	0.018^∧^	463.70 (391.08~570.35)	454.07 (325.93~575.84)	0.808	164.30 (103.70~253.35)	204.38 (135.11~261.23)	0.355
AUC_INS_/AUC_BG_	10.74 (5.51~18.15)	5.23 (3.11~10.49)	<0.001^∧^	11.51 (8.02~18.72)	5.85 (4.63~11.12)	0.001^∧^	16.30 (10.74~23.22)	9.08 (4.40~12.57)	<0.001^∧^	2.57 (1.23~6.34)	2.25 (1.56~4.61)	0.205

NGT: normal glucose tolerance; IGT: impaired glucose tolerance; DM: diabetes mellitus; IGF-1 index: the ratio of the measured IGF-1 value to the upper limit of normal (ULN); FPG: fasting plasma glucose; BG_120_: plasma glucose 120 min during OGTT; AUC_BG_: the areas under the curve of glucose; FPI: fasting plasma insulin; INS_120_: plasma insulin 120 min during OGTT; AUC_INS_: the areas under the curve of insulin; HOMA-IR: indicator of insulin resistance; IS_OGTT_: the OGTT insulin sensitivity index; HOMA-*β*: homeostatic model assessment of pancreatic beta-cell function; IGI: insulinogenic index; ISSI2: the OGTT insulin secretion sensitivity index. *p* values are for variations before and after SSA treatment; ^∧^ *p* < 0.05.

**Table 2:** Comparison of baseline characteristics of patients in the Improved/Stable/Deteriorated glucose tolerance status groups.

	Change in glucose status			*p* value
	Improved (*n* = 18)	Stable (*n* = 28)	Deteriorated (*n* = 18)	
Female [*n*/(%)]	11 (61.1)	17 (60.7)	10 (55.6)	0.927
Age (years)	38.4 ± 9.7	46.1 ± 13.2	38.1 ± 13.7	0.049^∧^
BMI (kg/m^2^)	21.99 ± 8.32	24.79 ± 6.26	24.99 ± 7.79	0.378
GH_m_ (*μ*g/l)	40.72 (25.88~86.15)	27.98 (15.69~70.23)	22.61 (12.65~49.69)	0.172
IGF-1 index	2.87 ± 1.05	2.94 ± 0.89	2.58 ± 0.65	0.489
HbA_1c_ (%)	5.80 (5.50~6.25)	6.30 (5.60~9.18)	5.70 (5.60~5.88)	0.196
FPG (mmol/l)	5.50 (5.18~6.18)	5.75 (5.03~6.70)	5.30 (5.10~5.80)	0.254
BG_120_ (mmol/l)	9.25 (8.68~12.73)	11.40 (7.83~17.13)	6.80 (5.58~8.23)	<0.001^∧^
FPI (mU/l)	17.65 (11.55~26.38)	13.02 (8.66~23.53)	16.40 (12.13~30.58)	0.329
INS_120_ (mU/l)	174.05 (63.53~248.98)	69.80 (25.18~152.33)	73.65 (48.90~189.85)	0.166

IGF-1 index: the ratio of the measured IGF-1 value to the upper limit of normal (ULN); HbA_1c_: glycosylated hemoglobin; FPG: fasting plasma glucose; BG_120_: plasma glucose 120 min during OGTT; FPI: fasting plasma insulin; INS_120_: plasma insulin 120 min during OGTT; *p* values are for variations among the 3 groups; ^∧^ *p* < 0.05.

**Table 3:** The change of glucose metabolism-related variables after SSA treatment among the Improved/Stable/Deteriorated groups.

Reduction (post-SSA)—basal	Improved (*n* = 18)	Stable (*n* = 28)	Deteriorated (*n* = 18)	*p* value
GH_m_ (*μ*g/l)	−28.01 (−53.71~−10.80)	−15.55 (−32.89~−6.50)	−5.89 (−16.55~−1.69)	0.021^∧^
IGF-1 index	−0.64 (−1.40~−0.26)	−1.16 (−1.44~−0.42)	−0.60 (−1.58~−0.18)	0.353
HOMA-IR	−0.75 (−4.15~0.00)	−1.45 (−2.98~−0.28)	−1.17 (−2.41~0.26)	0.830
IS_OGTT_	26.01 (5.16~54.78)	26.01 (5.23~53.45)	15.13 (0.54~51.43)	0.781
HOMA-*β* (%)	−56.15 (−103.77~−4.03)^∗^	−36.94 (−140.44~0.03)^∗^	−85.52 (−206.70~−65.53)	0.074
INS_0_/BG_0_	−0.78 (−2.65~−0.14)	−0.64 (−1.66~0.13)	−1.16 (−3.14~−0.81)	0.121
IGI/IR	−1.26 (−4.46~0.14)	−0.47 (−6.36~0.13)	−2.28 (−7.23~0.19)	0.844
ISSI2	36.16 (−118.62~113.34)	−15.97 (−59.98~54.94)	−84.97 (−158.72~100.10)	0.319
IGI	−17.10 (−28.02~−5.78)	−1.79 (−30.96~0.07)	−18.69 (−41.77~−5.24)	0.350
AUC_INS_/AUC_BG_	−4.76 (−10.44~−1.96)	−2.42 (−8.32~0.43)	−4.40 (−13.00~−1.93)	0.244

IGF-1 index: the ratio of the measured IGF-1 value to the upper limit of normal (ULN); HOMA-IR: indicator of insulin resistance; IS_OGTT_: the OGTT insulin sensitivity index; HOMA-*β*: homeostatic model assessment of pancreatic beta-cell function; INS_0_: fasting plasma insulin; BG_0_: fasting plasma glucose; IGI: insulinogenic index; AUC_BG_: the areas under the curve of glucose; AUC_INS_: the areas under the curve of insulin; ISSI2: the OGTT insulin secretion sensitivity index-2. *p* values are for variations before and after SSA treatment; ^∗^ *p* < 0.05 versus the Deteriorated group; ^∧^ *p* < 0.05.

## Supplementary Materials

Supplementary Materials Supplementary Table 1: baseline characteristics of the 64 patients. Supplementary Table 2: patients' data before and after SSA therapy. Supplementary Table 3: OGTT and glucose tolerance status before and after SSA therapy. Supplementary Table 4: insulin levels during OGTT before and after SSA therapy. Supplementary Table 5: comparison of pretreatment variables among NGT/IGT/DM groups. Supplementary Table 6: the baseline characteristics of the female group and male group. Supplementary Table 7: changes of variables in female and male groups from pretreatment to after SSA treatment. Supplementary Table 8: changes of variables in “controlled” and “uncontrolled” patients from pretreatment to after SSA treatment. Supplementary Table 9: correlation between the changes of HbA1c and glucose metabolism-related variables after SSA treatment. Supplementary Table 10: comparison of baseline characteristics between group A and B. Supplementary Table 11: comparison of the change in variables after SSA treatment between groups A and B.
