# Erratum to “Impact of Long-Acting Somatostatin Analogues on Glucose Metabolism in Acromegaly: A Hospital-Based Study”

**DOI:** 10.1155/2019/1987417

**Published:** 2019-03-10

**Authors:** Ming Shen, Meng Wang, Wenqiang He, Min He, Nidan Qiao, Zengyi Ma, Zhao Ye, Qilin Zhang, Yichao Zhang, Yeping Yang, Yanjiao Cai, Yakupujiang ABuDuoReYiMu, Yun Lu, Bin Lu, Xuefei Shou, Yongfei Wang, Hongying Ye, Yiming Li, Shiqi Li, Yao Zhao, Xiaoyun Cao, Zhaoyun Zhang

**Affiliations:** ^1^Department of Neurosurgery, Huashan Hospital, Fudan University, Shanghai 200040, China; ^2^Department of Endocrinology and Metabolism, Huashan Hospital, Fudan University, Shanghai 200040, China; ^3^Department of Endocrinology and Metabolism, The Second People's Hospital of Kashi, Xinjiang Uygur Autonomous Region 844000, China; ^4^Department of Nuclear Medicine, Huashan Hospital, Fudan University, Shanghai 200040, China

In the article titled “Impact of Long-Acting Somatostatin Analogues on Glucose Metabolism in Acromegaly: A Hospital-Based Study” [1], “IGT *N* = 2” in the Post SSA column in Figure 1 was incorrect due to a production error and should be “IGT *N* = 12”. The updated Figure 1 is as follows:

## Figures and Tables

**Figure 1:**
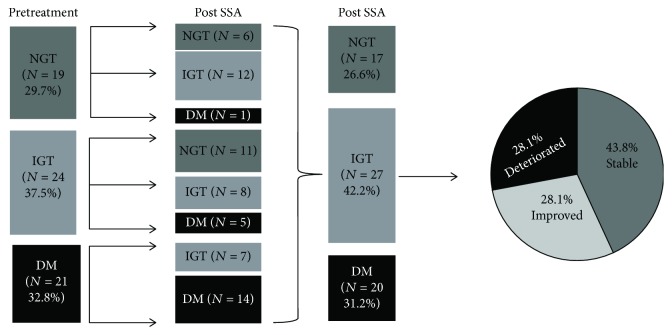
Flowchart of prevalence of NGT, IGT, and DM at pretreatment and after SSA treatment, and the change of glucose metabolism status after SSA therapy. NGT: normal glucose tolerance; IGT: impaired glucose tolerance; DM: diabetes mellitus.
