# Supplementary material for: Impact of Long-Acting Somatostatin Analogues on Glucose Metabolism in Acromegaly: A Hospital-Based Study
Source: Int J Endocrinol. 2018 Apr 26;2018:3015854. doi: 10.1155/2018/3015854 (PMC5944271; doi:10.1155/2018/3015854)
Supplement: Supplementary Materials — Supplementary Table 1: baseline characteristics of the 64 patients. Supplementary Table 2: patients' data before and after SSA therapy. Supplementary Table 3: OGTT and glucose tolerance status before and after SSA therapy. Supplementary Table 4: insulin levels during OGTT before and after SSA therapy. Supplementary Table 5: comparison of pretreatment variables among NGT/IGT/DM groups. Supplementary Table 6: the baseline characteristics of the female group and male group. Supplementary Table 7: changes of variables in female and male groups from pretreatment to after SSA treatment. Supplementary Table 8: changes of variables in “controlled” and “uncontrolled” patients from pretreatment to after SSA treatment. Supplementary Table 9: correlation between the changes of HbA1c and glucose metabolism-related variables after SSA treatment. Supplementary Table 10: comparison of baseline characteristics between group A and B. Supplementary Table 11: comparison of the change in variables after SSA treatment between groups A and B. [file 3015854.f1.docx]

**Supplementary Table 1** Baseline characteristics of the 64 patients.

|  | Value |
| --- | --- |
| Female [n/(%)] | 38 (59.4) |
| Age (years) | 41.7 ± 13.0 |
| BMI (kg/m^2^) | 25.70 ± 3.80 |
| GH_m_ (μg/L)  IGF-1 index | 31.42 (17.69 ~ 57.15)  2.71± 0.87 |
| HbA_1c_ (%) | 5.80 (5.60 ~ 6.40) |
| HOMA-IR | 4.27 (2.78 ~ 6.64) |
| HOMA-β (%) | 165.69 (83.85 ~ 255.24) |
| Glucose tolerance status |  |
| NGT [n/(%)] | 19 (29.7) |
| IGT [n/(%)] | 24 (37.5) |
| DM [n/(%)] | 21 (32.8) |

IGF-1 index, the ratio of the measured IGF-1 value to the upper limit of normal (ULN); HOMA-IR, indicator of insulin resistance; HOMA-β, homeostatic model assessment of pancreatic beta-cell function; NGT, normal glucose tolerance; IGT, impaired glucose tolerance; DM, diabetes mellitus. Data was expressed as mean ± SD or median with interquartile range.

**Supplementary Table 2** Patients’ data before and after SSA therapy.

| Patient: (no.) | Sex | Age (y) | BMI  (kg/m^2^) | SSA  * | GHm pre-SSA (μg/L) | GHm post- SSA (μg/L) | IGF-1 index pre- SSA | IGF-1 index post- SSA | HbA_1c_ pre- SSA (%) | HbA_1c_ post-  SSA (%) |
| --- | --- | --- | --- | --- | --- | --- | --- | --- | --- | --- |
| 1 | F | 51 | 23.70 | OCT | 17.73 | 2.18 | 4.98 | 1.12 | - | - |
| 2 | M | 51 | 25.60 | OCT | 31.42 | 8.74 | 2.71 | 2.07 | 13.60 | 6.10 |
| 3 | F | 38 | 27.30 | OCT | 123.24 | 4.33 | - | 2.19 | 12.40 | 6.70 |
| 4 | F | 26 | 22.20 | OCT | - | - | - | - | - | - |
| 5 | M | 24 | - | OCT | - | - | - | - | 5.80 | 6.00 |
| 6 | F | 27 | 23.00 | LAN | 86.15 | 91.38 | 2.35 | 2.09 | 5.10 | 5.80 |
| 7 | F | 47 | 24.09 | OCT | 20.78 | 3.59 | 2.37 | 1.60 | 5.60 | 5.60 |
| 8 | F | 33 | 22.10 | LAN | 39.24 | 48.45 | 2.84 | 2.25 | 5.70 | 7.20 |
| 9 | F | 27 | 24.20 | OCT | 132.69 | 1.11 | 3.07 | 0.61 | 5.70 | 5.50 |
| 10 | M | 48 | 25.14 | OCT | 22.75 | - | 1.60 | - | 9.70 | 6.10 |
| 11 | M | 17 | 35.50 | OCT | 49.69 | 69.46 | 2.15 | 1.04 | 5.60 | 6.30 |
| 12 | F | 60 | 26.20 | LAN | 13.88 | 7.38 | 2.27 | 1.96 | 9.70 | 8.80 |
| 13 | M | 35 | 26.00 | LAN | 27.98 | 14.54 | 3.83 | 3.67 | 10.70 | 8.00 |
| 14 | F | 67 | 28.00 | OCT | 89.62 | 12.91 | 2.44 | 1.63 | 6.40 | 6.50 |
| 15 | F | 59 | 24.65 | OCT | 77.31 | 76.92 | 3.25 | 2.89 | 6.40 | 6.20 |
| 16 | F | 41 | 24.10 | OCT | 33.79 | 7.92 | 3.70 | 2.52 | 7.10 | 7.00 |
| 17 | F | 44 | 22.60 | OCT | 40.72 | 3.59 | 2.66 | 1.62 | 6.40 | 6.20 |
| 18 | F | 43 | 23.40 | OCT | 16.70 | 17.18 | 2.70 | 2.49 | 5.90 | 6.50 |
| 19 | M | 59 | 27.00 | LAN | 12.65 | 1.16 | 3.19 | 1.13 | 5.80 | 5.80 |
| 20 | M | 56 | 27.70 | OCT | - | - | - | - | - | - |
| 21 | M | 65 | - | LAN | - | - | - | - | 5.60 | 5.60 |
| 22 | M | 42 | 22.00 | OCT | 43.50 | 10.61 | - | 2.22 | 7.30 | 7.00 |
| 23 | M | 61 | 28.00 | LAN | 35.71 | 26.37 | - | 2.92 | - | - |
| 24 | F | 30 | 36.50 | OCT | 22.61 | 7.59 | 3.06 | 2.46 | - | - |
| 25 | F | 31 | 32.00 | OCT | - | - | - | - | 6.00 | 6.40 |
| 26 | M | 47 | 32.33 | OCT | 23.27 | 2.56 | 4.63 | 3.14 | 6.20 | 5.30 |
| 27 | F | 34 | 22.65 | LAN | 60.89 | 4.25 | 2.04 | 0.64 | 5.70 | 5.50 |
| 28 | F | 34 | 26.10 | LAN | 25.88 | 15.54 | 1.68 | 1.73 | 6.10 | 6.10 |
| 29 | F | 17 | 28.34 | OCT | 10.50 | 6.13 | 1.18 | 1.35 | 5.70 | 7.00 |
| 30 | F | 19 | 21.67 | OCT | 114.08 | 79.69 | 1.70 | 1.33 | 5.60 | 5.70 |
| 31 | M | 34 | 30.19 | OCT | - | - | - | - | 6.30 | 6.10 |
| 32 | F | 32 | 19.00 | OCT | 8.80 | 1.10 | 1.66 | 0.68 | 5.70 | 6.00 |
| 33 | M | 39 | 26.40 | OCT | 94.77 | 1.44 | 4.99 | 0.65 | 5.60 | 5.50 |
| 34 | M | 23 | 24.40 | OCT | - | - | - | - | 5.70 | 5.70 |
| 35 | M | 36 | 25.62 | OCT | 54.62 | 47.46 | 3.13 | 3.21 | 5.80 | 5.30 |
| 36 | F | 38 | 24.54 | OCT | 21.81 | 2.57 | 3.11 | 1.31 | 5.50 | 5.50 |
| 37 | M | 41 | 26.51 | LAN | 70.23 | 6.10 | 3.40 | 1.96 | 5.60 | 5.50 |
| 38 | F | 33 | - | OCT | 126.00 | 5.33 | 3.73 | 1.28 | - | - |
| 39 | F | 30 | 23.14 | LAN | 24.42 | 23.05 | 2.02 | 1.88 | 5.70 | 5.90 |
| 40 | M | 54 | 29.36 | OCT | - | - | - | - | 5.40 | 5.50 |
| 41 | F | 59 | 30.41 | LAN | - | - | - | - | 10.60 | 9.30 |
| 42 | F | 54 | 37.65 | OCT | 6.54 | 2.60 | 3.18 | 2.24 | - | - |
| 43 | M | 44 | 24.69 | OCT | 15.69 | 0.65 | 2.19 | 0.76 | 5.00 | 5.00 |
| 44 | M | 48 | 26.99 | LAN | - | - | - | - | 6.90 | 6.70 |
| 45 | F | 14 | 24.68 | OCT | - | - | - | - | 5.50 | 6.00 |
| 46 | F | 33 | - | LAN | 56.15 | 15.04 | 1.95 | 2.68 | 5.10 | 5.10 |
| 47 | M | 44 | 18.94 | OCT | 3.73 | 1.08 | 3.30 | 1.23 | 5.40 | 5.60 |
| 48 | F | 39 | - | LAN | - | - | - | - | 5.80 | 5.30 |
| 49 | M | 51 | 23.18 | OCT | 37.35 | 4.01 | 4.95 | 3.23 | 6.00 | 6.10 |
| 50 | M | 44 | 21.78 | LAN | 21.25 | 4.19 | 3.25 | 2.19 | 5.50 | 5.70 |
| 51 | M | 50 | 22.15 | OCT | 32.67 | 20.48 | 2.95 | 2.25 | 5.60 | 5.80 |
| 52 | F | 50 | - | OCT | 40.27 | 34.05 | 2.35 | 2.11 | 5.20 | 5.60 |
| 53 | F | 54 | 25.71 | OCT | 21.08 | 3.15 | 2.50 | 2.50 | 9.00 | 6.70 |
| 54 | F | 50 | 23.53 | OCT | 17.65 | 0.41 | 2.41 | 1.13 | 8.10 | 7.10 |
| 55 | F | 40 | 24.44 | LAN | 18.32 | 1.72 | 3.04 | 2.74 | 5.60 | 5.90 |
| 56 | M | 38 | 23.29 | OCT | 46.46 | 1.56 | 3.20 | 1.80 | 5.50 | 5.40 |
| 57 | M | 46 | 26.30 | LAN | 30.21 | 1.56 | 2.76 | 1.62 | - | - |
| 58 | F | 49 | 24.97 | LAN | 43.25 | 0.78 | 2.61 | - | 6.10 | 5.50 |
| 59 | F | 40 | 24.56 | LAN | 76.46 | 23.34 | 2.56 | 2.33 | - | - |
| 60 | M | 47 | 31.22 | LAN | 92.46 | 0.71 | 3.06 | 2.42 | 6.00 | 5.70 |
| 61 | F | 14 | 24.17 | OCT | 8.61 | 2.55 | 1.45 | 1.01 | 5.90 | 6.20 |
| 62 | F | 56 | 24.67 | OCT | 5.68 | 0.12 | 3.11 | 0.75 | 5.80 | 6.00 |
| 63 | F | 48 | 23.42 | OCT | 5.87 | 0.44 | 2.01 | 0.78 | 5.00 | 5.10 |
| 64 | F | 63 | 22.48 | LAN | 2.58 | 1.68 | 2.66 | 1.34 | 9.90 | 7.20 |

*LAN: lanreotide SR, 40 mg/2 weeks ×12 weeks; OCT: octreotide LAR, 20 mg/4 weeks ×12 weeks.

**Supplementary Table 3** OGTT and glucose tolerance status before and after SSA therapy.

| Patient: (no.) | | BG_0_  pre-SSA  (mmol/l) | | BG_30_  pre-SSA  (mmol/l) | | BG_60_  pre-SSA  (mmol/l) | | BG_120_  pre-SSA  (mmol/l) | | BG_180_  pre-SSA  (mmol/l) | | Glucose tolerance status  pre-SSA | | BG_0_  post-SSA  (mmol/l) | | BG_30_  post-SSA  (mmol/l) | | BG_60_  post-SSA  (mmol/l) | | BG_120_  post-SSA  (mmol/l) | | BG_180_  post-SSA  (mmol/l) | | Glucose tolerance status  post-SSA | |
| --- | --- | --- | --- | --- | --- | --- | --- | --- | --- | --- | --- | --- | --- | --- | --- | --- | --- | --- | --- | --- | --- | --- | --- | --- | --- |
| 1 | 5.70 | | 9.60 | | 10.40 | | 8.80 | | 6.40 | | IGT | | 5.40 | | 7.10 | | 9.60 | | 10.90 | | 7.10 | | IGT | |  |
| 2 | 8.80 | | 17.60 | | 16.80 | | 17.60 | | 15.10 | | DM | | 6.30 | | 10.30 | | 12.90 | | 5.90 | | 5.30 | | IGT | |  |
| 3 | 6.40 | | 11.80 | | 16.20 | | 12.90 | | 10.50 | | DM | | 6.00 | | 8.80 | | 12.10 | | 8.30 | | 4.50 | | IGT | |  |
| 4 | 4.80 | | 7.60 | | 9.60 | | 8.40 | | 6.70 | | IGT | | 5.30 | | 6.00 | | 8.10 | | 7.40 | | 6.60 | | NGT | |  |
| 5 | 5.10 | | 7.40 | | 9.10 | | 5.50 | | 4.40 | | NGT | | 5.70 | | 7.80 | | 10.00 | | 7.60 | | 6.30 | | NGT | |  |
| 6 | 5.20 | | 6.50 | | 10.80 | | 11.80 | | 7.80 | | DM | | 5.40 | | 5.90 | | 8.90 | | 10.70 | | 8.10 | | IGT | |  |
| 7 | 6.30 | | 10.00 | | 12.30 | | 8.00 | | 7.20 | | IGT | | 6.00 | | 8.90 | | 13.00 | | 11.10 | | 4.90 | | DM | |  |
| 8 | 6.30 | | 10.70 | | 14.90 | | 16.60 | | 14.40 | | DM | | 7.40 | | 12.00 | | 17.00 | | 17.50 | | 14.60 | | DM | |  |
| 9 | 5.30 | | 9.00 | | 9.30 | | 9.80 | | 5.70 | | IGT | | 5.40 | | 5.90 | | 7.90 | | 8.20 | | 5.00 | | IGT | |  |
| 10 | 5.00 | | 6.30 | | 10.20 | | 13.90 | | 13.30 | | DM | | 5.60 | | 7.10 | | 10.80 | | 15.70 | | 12.20 | | DM | |  |
| 11 | 5.50 | | 7.70 | | 9.80 | | 5.90 | | 5.40 | | NGT | | 5.90 | | 7.20 | | 8.50 | | 8.00 | | 6.20 | | IGT | |  |
| 12 | 9.00 | | 15.00 | | 19.30 | | 20.30 | | 18.80 | | DM | | 12.40 | | 13.70 | | 19.90 | | 21.10 | | 19.60 | | DM | |  |
| 13 | 6.20 | | 11.10 | | 16.40 | | 14.60 | | 11.30 | | DM | | 4.10 | | 7.90 | | 11.90 | | 13.20 | | 25.80 | | DM | |  |
| 14 | 5.70 | | 9.80 | | 12.00 | | 15.50 | | 12.70 | | DM | | 4.60 | | 6.70 | | 11.80 | | 16.80 | | 14.50 | | DM | |  |
| 15 | 6.40 | | 11.60 | | 9.60 | | 6.50 | | 5.50 | | IGT | | 6.50 | | 10.40 | | 14.30 | | 10.20 | | 5.90 | | IGT | |  |
| 16 | 7.60 | | 11.70 | | 15.90 | | 17.30 | | 15.00 | | DM | | 7.20 | | 12.50 | | 14.60 | | 14.20 | | 11.30 | | DM | |  |
| 17 | 6.80 | | 13.40 | | 15.00 | | 11.30 | | 8.40 | | DM | | 6.30 | | 9.20 | | 11.60 | | 10.60 | | 8.10 | | IGT | |  |
| 18 | 5.30 | | 11.10 | | 10.20 | | 5.50 | | 4.10 | | NGT | | 6.80 | | 11.40 | | 15.30 | | 10.80 | | 8.70 | | IGT | |  |
| 19 | 5.40 | | 7.80 | | 9.80 | | 6.20 | | 6.20 | | NGT | | 5.90 | | 8.00 | | 12.20 | | 8.20 | | 3.80 | | IGT | |  |
| 20 | 6.10 | | 10.10 | | 10.70 | | 8.90 | | 5.00 | | IGT | | 7.10 | | 9.40 | | 10.30 | | 8.60 | | 7.90 | | DM | |  |
| 21 | 4.99 | | 8.17 | | 6.74 | | 4.76 | | 4.76 | | NGT | | 5.40 | | 9.70 | | 8.40 | | 7.40 | | 5.00 | | NGT | |  |
| 22 | 5.40 | | 11.20 | | 14.70 | | 12.60 | | 6.60 | | DM | | 5.10 | | 8.50 | | 12.20 | | 13.00 | | 10.10 | | DM | |  |
| 23 | 10.40 | | - | | - | | 19.50 | | - | | DM | | 10.60 | | 14.60 | | 18.20 | | 18.60 | | 17.90 | | DM | |  |
| 24 | 5.80 | | 8.50 | | 11.00 | | 9.60 | | 4.50 | | IGT | | 6.70 | | 9.30 | | 10.40 | | 11.50 | | 6.10 | | DM | |  |
| 25 | 5.10 | | 9.10 | | 10.80 | | 7.20 | | 5.50 | | NGT | | 6.60 | | 6.80 | | 9.00 | | 8.30 | | 8.50 | | IGT | |  |
| 26 | 4.78 | | - | | 12.09 | | 8.60 | | 5.86 | | IGT | | 4.70 | | 9.60 | | 10.80 | | 10.30 | | 5.50 | | IGT | |  |
| 27 | 5.10 | | 10.30 | | 11.30 | | 8.60 | | 3.90 | | IGT | | 5.20 | | 8.20 | | 11.40 | | 7.70 | | 7.30 | | NGT | |  |
| 28 | 5.60 | | 10.10 | | 11.20 | | 8.20 | | 7.20 | | IGT | | 5.50 | | 10.50 | | 10.60 | | 7.10 | | 7.20 | | NGT | |  |
| 29 | 4.40 | | 9.60 | | 11.70 | | 9.80 | | 6.20 | | IGT | | 7.60 | | 9.30 | | 12.10 | | 13.40 | | 12.60 | | DM | |  |
| 30 | 5.00 | | 10.60 | | 7.40 | | 7.70 | | 8.10 | | NGT | | 6.00 | | 7.00 | | 8.70 | | 8.70 | | 7.00 | | IGT | |  |
| 31 | 6.0 | | 7.20 | | 8.40 | | 9.20 | | 8.50 | | IGT | | 6.10 | | 9.10 | | 12.50 | | 12.50 | | 6.20 | | DM | |  |
| 32 | 4.70 | | 10.50 | | 11.60 | | 7.60 | | 5.20 | | NGT | | 5.20 | | 8.50 | | 12.00 | | 6.30 | | 3.40 | | NGT | |  |
| 33 | 5.70 | | 11.30 | | 12.30 | | 8.80 | | 5.90 | | IGT | | 5.10 | | 8.00 | | 9.10 | | 7.50 | | 5.20 | | NGT | |  |
| 34 | 5.20 | | 7.60 | | 6.90 | | 5.60 | | 4.80 | | NGT | | 5.20 | | 7.70 | | 8.10 | | 8.80 | | 5.90 | | IGT | |  |
| 35 | 5.20 | | 7.70 | | 10.30 | | 6.30 | | 4.30 | | NGT | | 5.20 | | 6.90 | | 8.80 | | 7.80 | | 4.90 | | IGT | |  |
| 36  37 | 5.50  5.90 | | 9.10  12.30 | | 9.20  13.30 | | 9.10  9.70 | | 4.10  7.00 | | IGT  IGT | | 5.60  6.50 | | 10.50  9.30 | | 7.20  11.90 | | 7.00  8.30 | | 3.50  8.70 | | NGT  IGT | |  |
| 38 | 6.30 | | 7.00 | | 12.40 | | 15.30 | | 15.60 | | DM | | 6.30 | | 8.10 | | 10.50 | | 12.00 | | 11.40 | | DM | |  |
| 39 | 5.00 | | 8.70 | | 8.90 | | 6.60 | | 4.80 | | NGT | | 5.50 | | 7.90 | | 10.20 | | 8.50 | | 7.40 | | IGT | |  |
| 40 | 4.90 | | 8.90 | | 8.40 | | 7.30 | | 7.10 | | NGT | | 5.10 | | 9.30 | | 12.20 | | 7.30 | | 5.90 | | NGT | |  |
| 41 | 5.50 | | 11.90 | | 14.50 | | 20.30 | | 20.30 | | DM | | 8.20 | | 13.40 | | 17.60 | | 18.40 | | 15.70 | | DM | |  |
| 42 | 6.20 | | 9.90 | | 10.00 | | 10.20 | | 6.20 | | IGT | | 6.40 | | 9.10 | | 12.30 | | 8.40 | | 5.90 | | IGT | |  |
| 43 | 3.80 | | 7.40 | | 10.20 | | 7.20 | | 3.20 | | NGT | | 4.40 | | 8.40 | | 5.90 | | 7.40 | | 4.90 | | NGT | |  |
| 44 | 6.00 | | 9.90 | | 14.50 | | 14.20 | | 8.80 | | DM | | 5.90 | | 12.40 | | 14.50 | | 9.70 | | 6.40 | | IGT | |  |
| 45 | 5.80 | | 10.60 | | 8.00 | | 8.50 | | 6.90 | | IGT | | 6.20 | | 7.80 | | 9.30 | | 8.70 | | 8.10 | | IGT | |  |
| 46 | 6.10 | | 7.30 | | 8.40 | | 8.20 | | 5.20 | | IGT | | 5.70 | | 8.10 | | 10.50 | | 6.40 | | 6.10 | | NGT | |  |
| 47 | 5.10 | | 9.60 | | 10.90 | | 4.90 | | 3.50 | | NGT | | 5.40 | | 8.90 | | 14.10 | | 8.70 | | 3.00 | | IGT | |  |
| 48 | 7.00 | | 7.90 | | 12.30 | | 12.70 | | 13.00 | | DM | | 6.50 | | 9.20 | | 11.00 | | 6.50 | | 6.30 | | NGT | |  |
| 49 | 5.50 | | 9.30 | | 10.20 | | 8.90 | | 4.80 | | IGT | | 6.00 | | 11.20 | | 12.90 | | 6.50 | | 6.30 | | NGT | |  |
| 50 | 5.10 | | 9.40 | | 9.60 | | 7.00 | | 5.20 | | NGT | | 5.80 | | 9.30 | | 11.40 | | 8.30 | | 5.50 | | IGT | |  |
| 51 | 4.80 | | 10.60 | | 11.30 | | 9.40 | | 3.80 | | IGT | | 5.30 | | 9.70 | | 11.20 | | 4.00 | | 4.30 | | NGT | |  |
| 52 | 5.40 | | 8.70 | | 8.80 | | 5.40 | | 4.20 | | NGT | | 6.20 | | 8.40 | | 10.40 | | 8.70 | | 5.60 | | IGT | |  |
| 53 | 13.10 | | 17.20 | | 20.00 | | 21.50 | | 19.60 | | DM | | 7.00 | | 9.00 | | 13.60 | | 15.10 | | 12.00 | | DM | |  |
| 54 | 6.80 | | 14.00 | | 16.40 | | 17.60 | | 12.20 | | DM | | 6.00 | | 10.30 | | 12.90 | | 14.20 | | 9.60 | | DM | |  |
| 55 | 5.50 | | 9.30 | | 13.00 | | 8.90 | | 5.50 | | IGT | | 5.90 | | 10.20 | | 14.70 | | 5.00 | | 4.60 | | NGT | |  |
| 56 | 4.80 | | 10.10 | | 12.70 | | 10.80 | | 7.00 | | IGT | | 5.60 | | 9.40 | | 8.50 | | 3.80 | | 4.30 | | NGT | |  |
| 57 | 4.70 | | 7.70 | | 6.00 | | 5.40 | | 5.60 | | NGT | | 5.50 | | 7.10 | | 8.50 | | 5.40 | | 5.50 | | NGT | |  |
| 58 | 5.80 | | 10.00 | | 11.70 | | 7.40 | | 2.80 | | NGT | | 5.70 | | 9.70 | | 12.80 | | 10.10 | | 3.70 | | IGT | |  |
| 59 | 8.30 | | 14.00 | | 17.10 | | 16.30 | | 11.00 | | DM | | 7.70 | | 11.60 | | 13.00 | | 13.80 | | 14.60 | | DM | |  |
| 60 | 5.30 | | 5.80 | | 9.00 | | 8.70 | | 7.30 | | IGT | | 5.30 | | 9.60 | | 10.10 | | 7.30 | | 4.70 | | NGT | |  |
| 61 | 5.40 | | 10.30 | | 11.10 | | 12.80 | | 9.50 | | DM | | 6.30 | | 11.40 | | 13.50 | | 9.30 | | 7.70 | | IGT | |  |
| 62 | 5.30 | | 8.90 | | 8.90 | | 4.90 | | 4.90 | | NGT | | 5.40 | | 6.40 | | 7.80 | | 12.10 | | 4.20 | | DM | |  |
| 63 | 5.20 | | 10.50 | | 11.10 | | 9.60 | | 7.00 | | IGT | | 6.20 | | 9.70 | | 14.20 | | 6.80 | | 7.90 | | IGT | |  |
| 64 | 11.80 | | 18.60 | | 21.10 | | 21.50 | | 16.20 | | DM | | 6.10 | | 9.80 | | 15.90 | | 20.00 | | 13.60 | | DM | |  |

NGT, normal glucose tolerance; IGT, impaired glucose tolerance; DM, diabetes mellitus.

**Supplementary Table 4** Insulin levels during OGTT before and after SSA therapy.

| Patient: (no.) | INS_0_  pre-SSA  (mU/l) | INS_30_  pre-SSA  (mU/l) | INS_60_  pre-SSA  (mU/l) | INS_120_  pre-SSA  (mU/l) | INS_180_  pre-SSA  (mU/l) | INS_0_  post-SSA  (mU/l) | INS_30_  post-SSA  (mU/l) | INS_60_  post-SSA  (mU/l) | INS_120_  post-SSA  (mU/l) | INS_180_  post-SSA  (mU/l) |
| --- | --- | --- | --- | --- | --- | --- | --- | --- | --- | --- |
| 1 | 21.50 | 209.00 | 246.00 | 209.90 | 80.20 | 6.20 | 13.30 | 19.90 | 64.60 | 36.40 |
| 2 | 4.20 | - | - | 8.80 | - | 3.70 | 11.00 | 21.70 | 11.00 | 8.10 |
| 3 | 73.90 | 80.20 | 83.00 | 103.30 | 88.90 | 29.10 | 31.80 | 48.60 | 71.10 | 38.10 |
| 4 | 36.90 | 145.60 | 230.40 | 300.00 | 300.00 | 17.90 | 33.00 | 53.80 | 120.70 | 98.40 |
| 5 | 49.90 | 242.90 | 300.00 | 155.80 | 92.50 | 25.90 | 127.70 | 167.50 | 166.90 | 171.90 |
| 6 | 21.10 | 50.50 | 148.60 | 294.20 | 224.30 | 20.00 | 22.60 | 60.30 | 136.30 | 71.70 |
| 7 | 12.50 | 58.40 | 97.70 | 80.10 | 95.90 | 8.70 | 28.60 | 44.60 | 186.10 | 44.20 |
| 8 | 67.60 | 120.60 | 142.50 | 259.70 | 300.00 | 23.70 | 34.60 | 54.80 | 112.30 | 100.40 |
| 9 | 25.60 | 191.30 | 181.50 | 300.00 | 105.30 | 3.50 | 6.50 | 14.40 | 61.20 | 22.10 |
| 10 | 3.40 | 5.30 | 3.40 | 24.30 | 19.30 | 1.80 | 2.70 | 10.80 | 2.20 | 31.50 |
| 11 | 87.10 | 290.70 | 300.00 | 300.00 | 161.10 | 65.00 | 126.60 | 173.20 | 216.60 | 188.00 |
| 12 | 6.00 | 12.40 | 12.90 | 23.20 | 19.80 | 4.00 | 9.00 | 9.40 | 9.60 | 7.70 |
| 13 | 12.40 | 13.40 | 16.20 | 22.30 | 15.90 | 10.20 | 12.60 | 18.90 | 36.20 | 24.90 |
| 14 | 14.60 | 25.40 | 39.80 | 68.70 | 91.90 | 7.90 | 10.30 | 13.00 | 33.30 | 35.80 |
| 15 | 11.00 | 300.00 | 42.90 | 141.90 | 10.60 | 11.40 | 60.90 | 300.00 | 283.50 | 55.30 |
| 16 | 13.30 | 20.70 | 39.70 | 44.40 | 53.10 | 6.00 | 21.30 | 26.60 | 39.00 | 33.00 |
| 17 | 13.50 | 63.30 | 18.60 | 60.30 | 37.10 | 3.70 | 7.60 | 17.20 | 31.90 | 20.50 |
| 18 | 14.10 | 91.40 | 53.60 | 44.60 | 3.30 | 11.80 | 26.20 | 53.40 | 74.90 | 52.90 |
| 19 | 4.90 | 53.80 | 124.00 | 104.40 | 53.30 | 3.40 | 17.70 | 54.50 | 287.70 | 18.10 |
| 20 | 10.79 | 46.74 | 49.33 | 40.02 | 16.32 | 6.80 | 17.50 | 21.10 | 18.50 | 11.90 |
| 21 | 8.04 | 178.30 | 4.83 | 21.20 | 7.39 | 8.70 | 3.10 | 215.50 | 63.30 | 15.50 |
| 22 | 0.50 | 19.40 | 15.70 | 10.70 | 23.40 | 3.30 | 8.90 | 10.80 | 18.30 | 48.70 |
| 23 | 20.10 | - | - | 44.60 | - | 20.20 | 2.80 | 1.00 | 1.60 | 8.30 |
| 24 | 29.40 | 81.80 | 172.60 | 181.20 | 58.70 | 27.90 | 74.90 | 86.50 | 203.00 | 50.80 |
| 25 | 60.00 | 300.00 | 300.00 | 300.00 | 239.70 | 42.40 | 108.60 | 108.90 | 131.20 | 120.00 |
| 26 | 13.13 | - | 99.66 | 78.26 | 34.35 | 6.90 | 31.60 | 33.30 | 43.30 | 21.10 |
| 27 | 14.20 | 133.80 | 182.70 | 161.20 | 29.40 | 10.10 | 27.80 | 49.10 | 40.30 | 37.90 |
| 28 | 16.60 | 195.00 | 169.00 | 167.80 | 76.50 | 10.20 | 60.60 | 76.50 | 98.50 | 37.60 |
| 29 | 47.80 | 300.00 | 300.00 | 300.00 | 290.40 | 49.00 | 47.50 | 82.50 | 158.70 | 147.70 |
| 30 | 15.70 | 300.00 | 62.00 | 111.40 | 170.90 | 16.00 | 0.90 | 1.90 | 138.10 | 4.60 |
| 31 | 17.10 | 26.00 | 37.70 | 50.30 | 72.3 | 11.90 | 40.30 | 89.50 | 194.90 | 36.00 |
| 32 | 2.70 | 33.80 | 74.40 | 66.90 | 36.30 | 3.80 | 16.90 | 36.10 | 63.10 | 33.70 |
| 33 | 19.70 | 124.30 | 178.70 | 180.30 | 60.30 | 2.00 | 9.80 | 26.10 | 22.60 | 10.70 |
| 34 | 14.00 | 138.80 | 42.50 | 44.70 | 15.30 | 6.70 | 35.10 | 32.40 | 44.70 | 41.60 |
| 35 | 24.10 | 137.70 | 210.30 | 67.20 | 28.80 | 8.40 | 49.40 | 58.00 | 63.90 | 17.10 |
| 36 | 25.40 | 196.50 | 72.00 | 187.50 | 32.70 | 5.50 | 108.70 | 72.20 | 44.50 | 5.60 |
| 37  38 | 16.90  12.40 | 149.10  30.30 | 168.90  59.10 | 106.70  109.90 | 37.40  103.10 | 11.00  6.60 | 24.00  12.40 | 40.30  18.80 | 32.70  36.80 | 52.50  24.30 |
| 39 | 34.10 | 300.00 | 300.00 | 148.90 | 51.70 | 18.20 | 60.20 | 90.10 | 123.70 | 108.10 |
| 40 | 12.70 | 149.50 | 141.60 | 70.90 | 51.00 | 4.20 | 24.10 | 36.80 | 46.40 | 26.20 |
| 41 | 42.30 | 61.20 | 76.80 | 84.40 | 85.80 | 65.60 | 70.70 | 102.90 | 104.70 | 92.80 |
| 42 | 24.20 | 1.80 | 1.90 | 192.30 | 51.00 | 16.60 | 48.20 | 80.80 | 59.00 | 23.00 |
| 43 | 3.80 | 64.90 | 87.30 | 62.30 | 15.90 | 4.40 | 13.90 | 26.90 | 41.90 | 14.90 |
| 44 | 10.80 | 15.60 | 26.80 | 25.40 | 30.60 | 9.30 | 29.10 | 42.00 | 39.00 | 21.00 |
| 45 | 96.50 | 300.00 | 293.22 | 300.00 | 300.00 | 49.10 | 130.20 | 85.20 | 204.10 | 165.60 |
| 46 | 53.50 | 154.30 | 221.50 | 300.00 | 84.90 | 35.70 | 163.40 | 258.50 | 217.00 | 112.80 |
| 47 | 9.70 | 61.90 | 153.40 | 60.20 | 10.40 | 2.80 | 16.20 | 39.00 | 97.20 | 10.50 |
| 48 | 24.80 | 37.50 | 69.70 | 108.70 | 95.40 | 12.80 | 18.50 | 40.90 | 27.90 | 20.40 |
| 49 | 14.10 | 236.10 | 277.10 | 233.90 | 27.30 | 11.20 | 121.60 | 195.10 | 51.70 | 22.40 |
| 50 | 11.00 | 151.90 | 39.40 | 62.40 | 3.10 | 7.50 | 35.20 | 68.00 | 64.50 | 12.90 |
| 51 | 11.80 | 201.40 | 186.90 | 201.50 | 33.30 | 9.20 | 48.50 | 300.00 | 2.90 | 22.70 |
| 52 | 22.50 | 77.10 | 106.20 | 58.90 | 26.80 | 20.80 | 21.90 | 41.90 | 115.00 | 26.40 |
| 53 | 10.50 | 19.30 | 19.80 | 17.10 | 12.50 | 22.40 | 26.50 | 50.80 | 61.00 | 81.10 |
| 54 | 4.90 | 16.10 | 25.20 | 38.20 | 37.10 | 6.80 | 10.00 | 16.30 | 20.20 | 16.70 |
| 55 | 29.30 | 123.50 | 300.00 | 300.00 | 300.00 | 47.70 | 3.20 | 260.20 | 300.00 | 124.00 |
| 56 | 9.10 | 65.80 | 63.30 | 64.60 | 49.00 | 8.80 | 25.00 | 44.10 | 11.80 | 6.30 |
| 57 | 12.90 | 247.00 | 42.50 | 27.80 | 32.40 | 12.60 | 19.00 | 50.40 | 8.20 | 4.20 |
| 58 | 23.30 | 152.20 | 169.10 | 215.80 | 50.30 | 7.00 | 44.00 | 57.20 | 88.30 | 15.90 |
| 59 | 17.70 | 57.40 | 125.70 | 120.30 | 58.50 | 10.00 | 39.00 | 37.80 | 64.90 | 49.80 |
| 60 | 18.70 | 29.30 | 155.70 | 184.00 | 161.50 | 20.10 | 88.20 | 152.00 | 72.00 | 31.70 |
| 61 | 2.67 | 5.67 | 8.17 | 14.00 | 14.07 | 37.10 | 89.40 | 148.60 | 153.20 | 111.80 |
| 62 | 15.00 | 126.50 | 166.40 | 30.30 | 26.30 | 5.70 | 8.50 | 11.80 | 56.80 | 9.60 |
| 63 | 25.30 | 207.40 | 121.60 | 293.22 | 176.80 | 7.40 | 40.80 | 184.20 | 153.30 | 111.50 |
| 64 | 11.40 | 19.10 | 17.40 | 20.70 | 15.60 | 14.80 | 18.80 | 23.50 | 28.60 | 25.70 |

**Supplementary Table 5** Comparison of pre-treatment variables among NGT / IGT / DM groups.

|  | NGT (n = 19) | IGT (n = 24) | DM (n = 21) | *P* value |
| --- | --- | --- | --- | --- |
| Female [n/(%)] | 8 (42.1) | 15 (62.5) | 15 (71.4) | 1.000 |
| Age (years) | 40.3 ± 13.9 | 39.8 ± 11.8 | 45.1 ± 13.1 | 0.339 |
| BMI (kg/m^2^) | 25.15 ± 4.36 | 26.53 ± 4.35 | 25.15 ± 2.32 | 0.366 |
| GH_m_ (μg/L) | 22.84 (11.69 ~ 44.86) | 32.67 (19.55 ~ 65.56) | 34.75 (20.22 ~ 78.88) | 0.348 |
| IGF-1 index | 2.58 ± 0.57 | 3.10 ± 1.10 | 2.65 ± 0.69 | 0.153 |
| HbA_1c_ (%) | 5.64 ± 0.27 | 5.72 ± 0.37 | 8.35 ± 2.47 | < 0.001^^^ |
| HOMA-IR | 3.32 (2.20 ~ 6.01) | 4.78 (3.28 ~ 7.04) | 4.08 (2.20 ~ 7.13) | 0.286 |
| HOMA-β (%) | 202.61 (137.50 ~283.53) | 188.50 (140.87 ~ 290.86) | 73.75 (27.79 ~ 137.22) | 0.001^^^ |

NGT, normal glucose tolerance; IGT, impaired glucose tolerance; DM, diabetes mellitus; IGF-1 index, the ratio of the measured IGF-1 value to the upper limit of normal (ULN); HbA_1c_, glycosylated hemoglobin; HOMA-IR, indicator of insulin resistance; HOMA-β, homeostatic model assessment of pancreatic beta-cell function; *P* values are for variations among the 3 groups; ^^^ *P* < 0.05. Data was expressed as mean ± SD or median with interquartile range.

**Supplementary Table 6 The baseline characteristics of Female group and Male group.**

| Baseline | Females (n=38) | Males (n=25) | P value |
| --- | --- | --- | --- |
| Age (years) | 40.11±13.75 | 44.00±11.44 | 0.239 |
| BMI (kg/m^2^) | 25.24±3.77 | 26.39±3.67 | 0.239 |
| HbA_1c_ (%) | 6.70±1.94 | 6.42±1.96 | 0.579 |
| GH_m_ (μg/L) | 25.15 (17.41 ~ 76.67) | 32.05 (21.63 ~ 48.88) | 0.654 |
| IGF-1 index | 2.65±0.80 | 3.13±0.86 | 0.033^^^ |
| FPG (mmol/l) | 6.22±1.77 | 5.60±1.31 | 0.132 |
| BG_120_ (mmol/l) | 11.27±4.75 | 9.29±3.94 | 0.085 |
| FPI (mU/l) | 21.30 (13.10 ~ 30.58) | 12.55 (8.84 ~ 17.50) | 0.003^^^ |
| INS_120_ (mU/l) | 131.10 (59.95 ~ 268.08) | 62.35 (27.20 ~ 118.98) | 0.011^^^ |
| HOMA-IR | 5.90 (3.50 ~ 7.63) | 2.85 (1.88 ~ 4.45) | 0.02^^^ |
| IS_OGTT_ | 31.30 (22.18 ~ 47.96) | 66.80 (35.50 ~ 82.50) | 0.01^^^ |
| HOMA-β (%) | 187.40 (80.33 ~ 326.08) | 140.40 (85.55 ~ 205.85) | < 0.001^ |
| INS_0_/BG_0_ | 3.65 (2.00 ~ 5.68) | 2.35 (1.75 ~ 2.90) | 0.012^^^ |
| IGI | 21.00 (2.90 ~ 43.00) | 20.70 (9.00 ~ 52.00) | 0.521 |
| IGI/IR | 3.75 (0.93 ~ 7.40) | 7.40 (3.70 ~ 17.00) | 0.185 |
| ISSI2 | 425.20 (183.98 ~ 531.33) | 553.90 (354.00 ~ 710.90) | 0.821 |
| AUC_INS_/AUC_BG_ | 12.90 (5.65 ~ 20.83) | 10.20 (5.30 ~ 14.70) | 0.821 |

IGF-1 index, the ratio of the measured IGF-1 value to the upper limit of normal (ULN); HOMA-IR, indicator of insulin resistance; HOMA-β, homeostatic model assessment of pancreatic beta-cell function;.AUC_BG_, the areas under the curve of glucose; AUC_INS_, the areas under the curve of insulin; IS_OGTT_, the OGTT insulin sensitivity index; IGI, insulinogenic index; ISSI2, the OGTT insulin secretion sensitivity index. *P* values are for variations before and after SSA treatment; ^^^ *P* < 0.05.

**Supplementary Table 7** Changes of variables in Female and Male group from pre-treatment to after SSA treatment.

|  | Females (n = 38) | | | Males (n = 26) | | |
| --- | --- | --- | --- | --- | --- | --- |
|  | Pre-SSA | Post-SSA | P value | Pre-SSA | Post-SSA | P value |
| HOMA-IR | 5.90 (3.50 ~ 7.63) | 3.65 (1.80 ~ 7.80) | 0.008^ | 2.85 (1.88 ~ 4.45) | 2.00 (0.98 ~ 3.13) | 0.001^ |
| IS_OGTT_ | 31.30 (22.18 ~ 47.96) | 51.65 (30.30 ~ 103.62) | < 0.001^ | 66.80 (35.50 ~ 82.50) | 94.60 (64.51 ~ 148.85) | 0.003^ |
| HOMA-β (%) | 187.40 (80.33 ~ 326.08) | 114.15 (54.70 ~ 201.80) | < 0.001^ | 140.40 (85.55 ~ 205.85) | 81.30 (40.43 ~ 105.40) | 0.001^ |
| INS_0_/BG_0_ | 3.65 (2.00 ~ 5.68) | 1.95 (1.10 ~ 3.83) | < 0.001^ | 2.35 (1.75 ~ 2.90) | 1.60 (0.75 ~ 1.93) | < 0.001^ |
| IGI | 21.00 (2.90 ~ 43.00) | 4.10 (1.25 ~ 10.65) | < 0.001^ | 20.70 (9.00 ~ 52.00) | 4.65 (2.25 ~ 9.98) | < 0.001^ |
| IGI/IR | 3.75 (0.93 ~ 7.40) | 1.85 (0.38 ~ 3.93) | 0.012^ | 7.40 (3.70 ~ 17.00) | 2.86 (1.43 ~ 5.76) | 0.003^ |
| ISSI2 | 425.20 (183.98 ~ 531.33) | 318.10 (202.73 ~ 493.90) | < 0.001^ | 553.90 (354.00 ~ 710.90) | 444.45 (301.00 ~ 644.70) | 0.627 |
| AUC_INS_/AUC_BG_ | 12.90 (5.65 ~ 20.83) | 5.90 (3.55 ~ 11.02) | 0.243 | 10.20 (5.30 ~ 14.70) | 4.00 (2.28 ~ 10.38) | 0.002^ |

AUC_BG_, the areas under the curve of glucose; AUC_INS_, the areas under the curve of insulin; HOMA-IR, indicator of insulin resistance; IS_OGTT_, the OGTT insulin sensitivity index; HOMA-β, homeostatic model assessment of pancreatic beta-cell function; IGI, insulinogenic index; ISSI2, the OGTT insulin secretion sensitivity index. *P* values are for variations before and after SSA treatment; ^^^ *P* < 0.05.

**Supplementary Table 8** Changes of variables in “controlled” and “uncontrolled” patients from pre-treatment to after SSA treatment.

|  | controlled group (n = 16) | | | uncontrolled group (n = 35) | | |
| --- | --- | --- | --- | --- | --- | --- |
|  | Pre-SSA | Post-SSA | P value | Pre-SSA | Post-SSA | P value |
| HbA_1c_ (%) | 6.09±1.32 | 5.81±0.64 | 0.206 | 6.28±1.77 | 6.34±1.55 | 0.149 |
| FPG (mmol/l) | 5.68±1.75 | 5.53±0.45 | 0.706 | 5.68±1.75 | 5.53±0.45 | 0.826 |
| BG_120_ (mmol/l) | 8.75 (6.45 ~ 9.75) | 7.85 (6.42 ~ 10.70) | 0.649 | 9.70 (8.00 ~ 15.30) | 10.20 (8.00 ~ 13.20) | 0.417 |
| HOMA-IR | 3.95 (1.60 ~ 5.95) | 1.65 (0.90 ~ 2.88) | 0.153 | 4.10 (3.20 ~ 6.70) | 3.20 (1.90 ~ 7.00) | 0.003^ |
| IS_OGTT_ | 50.45 (26.20 ~ 84.10) | 121.85 (67.23 ~ 154.40) | 0.001^ | 39.55 (26.80 ~ 49.43) | 62.00 (39.70 ~ 96.20) | 0.002^ |
| HOMA-β (%) | 187.30 (69.03 ~ 243.73) | 61.80 (38.78 ~ 109.80) | 0.015^ | 156.67 (75.90 ~ 254.00) | 102.20 (56.90 ~ 174.40) | < 0.001^ |
| INS_0_/BG_0_ | 2.75 (1.00 ~ 3.95) | 1.10 (0.63 ~ 2.13) | 0.073 | 2.70 (2.00 ~ 4.60) | 1.90 (1.30 ~ 3.30) | < 0.001^ |
| IGI | 20.80 (10.93 ~ 33.55) | 4.00 (2.48 ~ 6.60) | 0.001^ | 20.05 (2.78 ~ 46.98) | 5.00 (1.60 ~ 11.70) | < 0.001^ |
| IGI/IR | 5.70 (3.98 ~ 9.33) | 3.05 (1.45 ~ 5.58) | 0.006^ | 4.45 (0.78 ~ 9.18) | 1.80 (0.70 ~ 3.90) | 0.005^ |
| ISSI2 | 547.80 (449.83 ~ 721.85) | 487.40 (407.35 ~ 797.58) | 0.756 | 416.25 (189.60 ~ 523.43) | 327.80 (204.40 ~ 479.20) | 0.217 |
| AUC_INS_/AUC_BG_ | 11.70 (6.73 ~ 20.28) | 4.10 (3.10 ~ 9.50) | 0.002^ | 10.30 (4.73 ~ 16.60) | 5.90 (2.30 ~ 9.40) | < 0.001^ |

AUC_BG_, the areas under the curve of glucose; AUC_INS_, the areas under the curve of insulin; HOMA-IR, indicator of insulin resistance; IS_OGTT_, the OGTT insulin sensitivity index; HOMA-β, homeostatic model assessment of pancreatic beta-cell function; IGI, insulinogenic index; ISSI2, the OGTT insulin secretion sensitivity index. *P* values are for variations before and after SSA treatment; ^^^ *P* < 0.05.

**Supplementary Table 9** Correlation between the changes of HbA1c and glucose metabolism-related variables after SSA treatment.

| Reduction (%) | HbA_1c_ | |
| --- | --- | --- |
|  | *r* | *P value* |
| GH_m_ | 0.348 | 0.018^^^ |
| IGF-1 index | 0.088 | 0.583 |
| HOMA-IR | 0.150 | 0.274 |
| IS_OGTT_ | -0.035 | 0.806 |
| HOMA-β | -0.240 | 0.077 |
| INS_0_/BG_0_ | -0.120 | 0.381 |
| IGI | -0.294 | 0.032^^^ |
| IGI/IR | -0.273 | 0.048^^^ |
| ISSI2 | -0.408 | 0.003^^^ |
| AUC_INS_/AUC_BG_ | -0.224 | 0.107 |

IGF-1 index, the ratio of the measured IGF-1 value to the upper limit of normal (ULN); HOMA-IR, indicator of insulin resistance; IS_OGTT_, the OGTT insulin sensitivity index; HOMA-β, homeostatic model assessment of pancreatic beta-cell function; IGI, insulinogenic index; ISSI2, the OGTT insulin secretion sensitivity index-2; AUC_BG_, the areas under the curve of glucose; AUC_INS_, the areas under the curve of insulin. ^^^ *P* < 0.05.

**Supplementary Table 10** Comparison of baseline characteristics between group A and B.

| Baseline | Group A | Group B | *P* value |
| --- | --- | --- | --- |
|  | BG_120_ > 8.1mmol/l | BG_120_ < 8.1mmol/l |  |
| GH_m_ (μg/L) | 33.79 (19.70 ~ 65.56) | 22.84 (13.41 ~ 48.08) | 0.208 |
| IGF-1 index | 2.84 (2.31 ~ 3.30) | 2.66 (2.16 ~ 3.18) | 0.509 |
| HOMA-IR | 4.56 (2.93 ~ 7.16) | 3.32 (2.35 ~ 5.79) | 0.143 |
| IS_OGTT_ | 34.31 (24.54 ~ 54.09) | 50.78 (31.81 ~ 69.31) | 0.172 |
| HOMA-β (%) | 141.71 (73.75 ~ 255.65) | 181.43 (114.59 ~ 268.43) | 0.268 |
| INS_0_/BG_0_ | 2.78 (1.80 ~ 4.83) | 2.69 (1.81 ~ 4.40) | 0.954 |
| IGI | 13.08 (1.89 ~ 33.94) | 34.20 (16.76 ~ 57.79) | 0.001^^^ |
| IGI/IR | 2.82 (0.66 ~ 5.86) | 9.48 (4.76 ~ 16.70) | < 0.001^^^ |
| ISSI2 | 320.96 (168.83 ~ 516.73) | 586.01 (486.77 ~ 690.60) | < 0.001^^^ |
| AUC_INS_/AUC_BG_ | 7.70 (2.84 ~ 18.90) | 11.51 (8.28 ~ 17.69) | 0.133 |

IGF-1 index, the ratio of the measured IGF-1 value to the upper limit of normal (ULN); HOMA-IR, indicator of insulin resistance; IS_OGTT_, the OGTT insulin sensitivity index; HOMA-β, homeostatic model assessment of pancreatic beta-cell function; IGI, insulinogenic index; ISSI2, the OGTT insulin secretion sensitivity index-2; AUC_BG_, the areas under the curve of glucose; AUC_INS_, the areas under the curve of insulin. ^^^ *P* < 0.05.

**Supplementary Table 11** Comparison of the change in variables after SSA treatment between group A and B.

| Reduction | Group A | Group B | *P* value |
| --- | --- | --- | --- |
| (Post-SSA) - Basal | BG_120_ > 8.1mmol/l | BG_120_ < 8.1mmol/l |  |
| GH_m_ (μg/L) | -17.59 (-46.96 ~ -6.39) | -7.16 (-17.13 ~ -0.88) | 0.019^^^ |
| IGF-1 index | -0.81 (-1.42 ~ -0.28) | -0.98 (-1.43 ~ -0.24) | 0.938 |
| HOMA-IR | -1.33 (-3.66 ~ 0.23) | -1.16 (-2.65 ~ 0.27) | 0.622 |
| IS_OGTT_ | 25.80 (4.70 ~ 50.76) | 17.01 (1.98 ~ 52.54) | 0.721 |
| HOMA-β (%) | -55.39 (-130.19 ~ -1.36) | -89.00 (-170.14 ~ -47.77) | 0.037^^^ |
| INS_0_/BG_0_ | -0.84 (-2.51 ~ -0.03) | -0.93 (-2.91 ~ -0.39) | 0.371 |
| IGI | -5.78 (-23.41~ -0.06) | -24.85 (-44.02 ~ -11.89) | 0.002^^^ |
| IGI/IR | -0.36 (-3.56 ~ 0.26) | -5.55 (-11.80 ~ -0.31) | 0.008^^^ |
| ISSI2 | -10.28 (-82.25 ~ 77.19) | -61.13 (-165.27 ~ 48.42) | 0.046^^^ |
| AUC_INS_/AUC_BG_ | -2.99 (-8.91 ~ -0.44) | -4.94 (-10.11 ~ -1.31) | 0.439 |

IGF-1 index, the ratio of the measured IGF-1 value to the upper limit of normal (ULN); HOMA-IR, indicator of insulin resistance; IS_OGTT_, the OGTT insulin sensitivity index; HOMA-β, homeostatic model assessment of pancreatic beta-cell function; IGI, insulinogenic index; ISSI2, the OGTT insulin secretion sensitivity index-2; AUC_BG_, the areas under the curve of glucose; AUC_INS_, the areas under the curve of insulin. ^^^ *P* < 0.05.
